# Home at last: the enigmatic genera *Eriachaenium* and *Adenocaulon* (Compositae, Mutisioideae, Mutisieae, Adenocaulinae)

**DOI:** 10.3897/phytokeys.60.6795

**Published:** 2016-02-11

**Authors:** Vicki A. Funk, Eduardo Pasini, J. Mauricio Bonifacino, Liliana Katinas

**Affiliations:** 1 Department of Botany, NMNH, Smithsonian Institution, Washington D.C., USA; 2 Universidade Federal do Rio Grande do Sul, Programa de Pós-Graduação em Botânica, Av. Bento Gonçalves 9500, CEP 91501-970, Porto Alegre, RS, Brazil; 3 Laboratorio de Botánica, Facultad de Agronomía, Universidad de la República, Av. Garzón 780, Sayago, Montevideo, CP, 12900, Uruguay; 4 División Plantas Vasculares, Museo de La Plata, Paseo del Bosque s/n, CP 1900, La Plata, Argentina

**Keywords:** Asteraceae, dimorphic flowers, endemism, Patagonia, Asia-North America disjunct

## Abstract

The genera *Eriachaenium* and *Adenocaulon* (Compositae) have distinct but complex histories and both have been placed in a number of tribes across the family. For the first time the two genera are included in a molecular study and the results show that they are best placed in the tribe Mutisieae s.s. and are the only genera in the re-instated subtribe Adenocaulinae. When described, this subtribe contained only *Adenocaulon* and was found in the Inuleae. The study also confirms one of the conclusions of a recent morphological study that *Eriachaenium* and *Adenocaulon* are sister taxa. Past difficulties in tribal assignment are attributed to the distinct and unusual morphology of each genus. Both genera and the subtribe are described and a key to separate the genera is provided.

## Introduction

**Figure 1.**
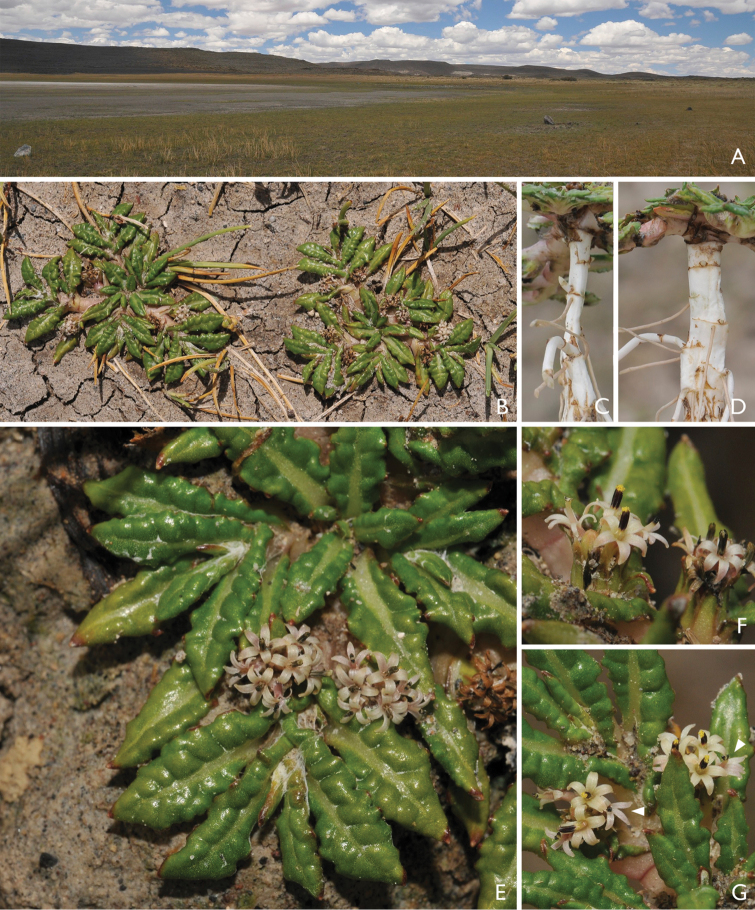
*Eriachaenium*. **A** General habitat, muddy shores of temporal lakes; central Chubut (Argentina) **B** Habit detail; notice stems adpressed to the ground **C** Lateral view of rhizome **D** Dorsal view of rhizome; compare with C and note the flattened nature of rhizome **E** Close up of leaves, note the bullate condition. **F** Detail of heads **G** Detail of heads; arrows point marginal florets. (Photos by M. Bonifacino)

**Figure 2.**
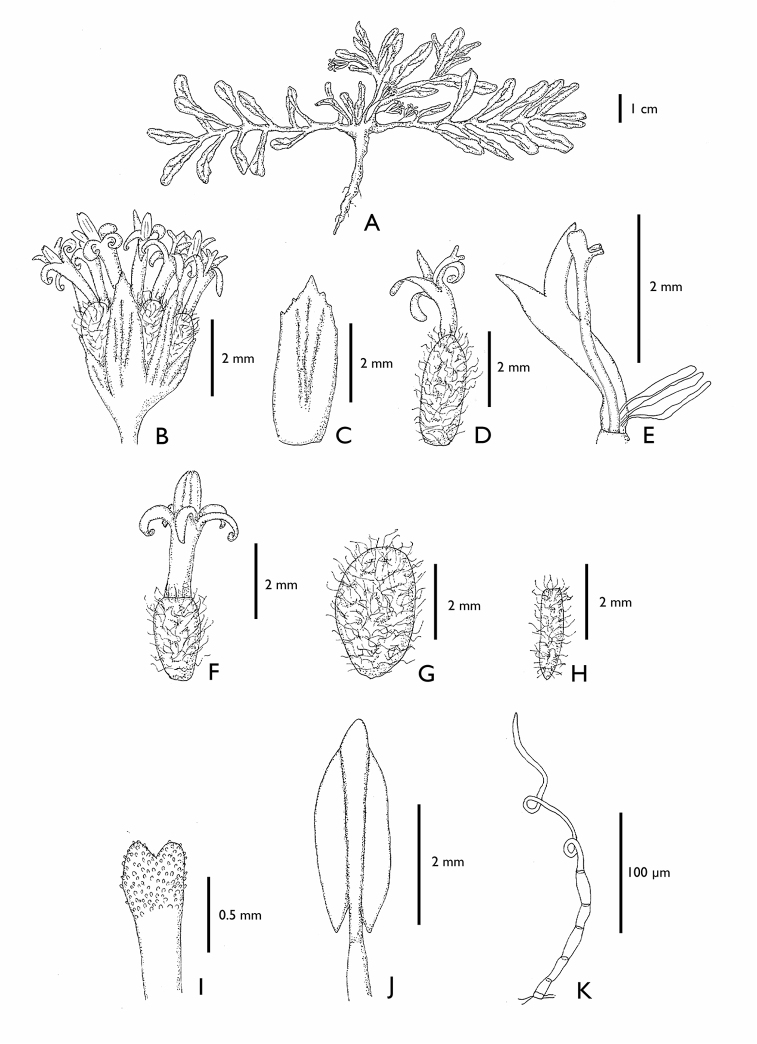
*Eriachaenium magellanicum* (from Katinas 2000). **A** habit **B** head **C** involucral bract **D** marginal floret **E** marginal floret opened showing the staminodes **F** central floret **G** marginal achene **H** central achene **I** upper part of style **J** stamen **K** cypsela hair flagellate, filiform. (*Eriachaenium magellanicum*: **A** *Birabén and Birabén 242* LP; **B–F, I–K** *Sleumer 908* LP; **G–H** LP *s.n. ex* LPS *13745*)

**Figure 3.**
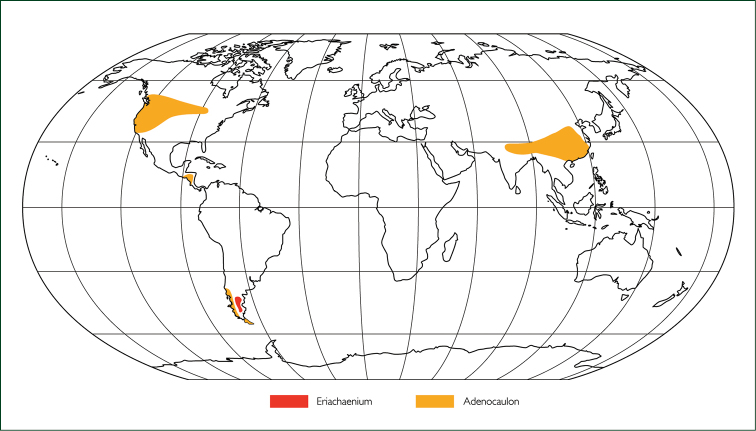
Map showing distribution of *Eriachaenium* and *Adenocaulon*.

**Figure 4.**
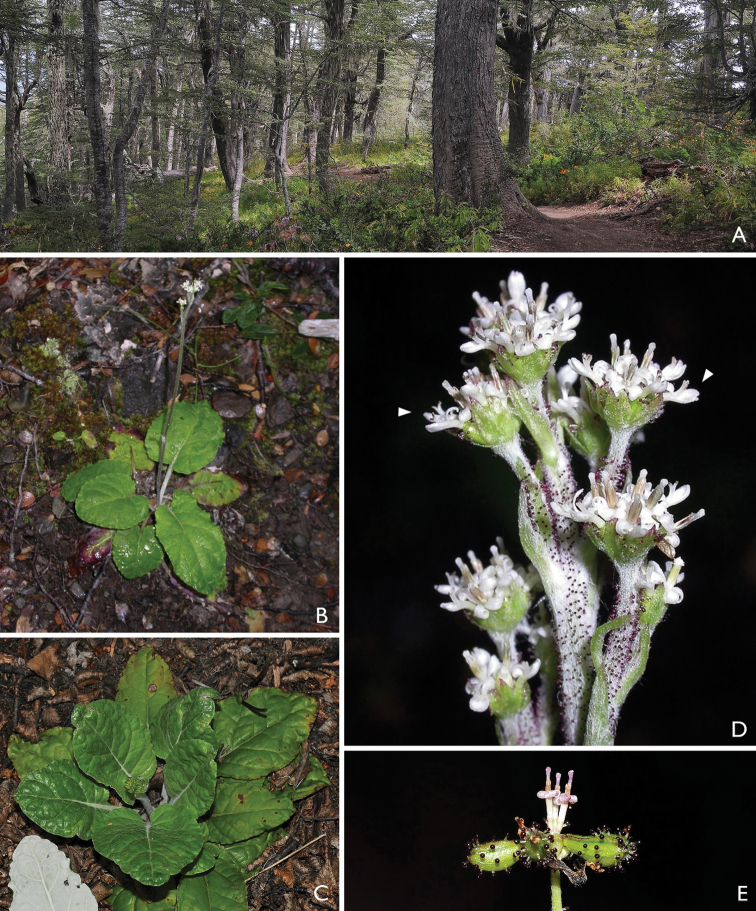
*Adenocaulon*. **A** Habitat, understory of *Nothofagus* dominated forest (Araucanía Region, Chile) **B** Habit **C** Close up of rosette, note the bullate leaves **D** Close up of heads; arrows indicate marginal florets, note the conspicuous glandular trichomes on peduncles and other parts of the inflorescence **E** Close up of fruiting head; note the same trichomes on fruits. (Photos by M. Bonifacino)

**Figure 5.**
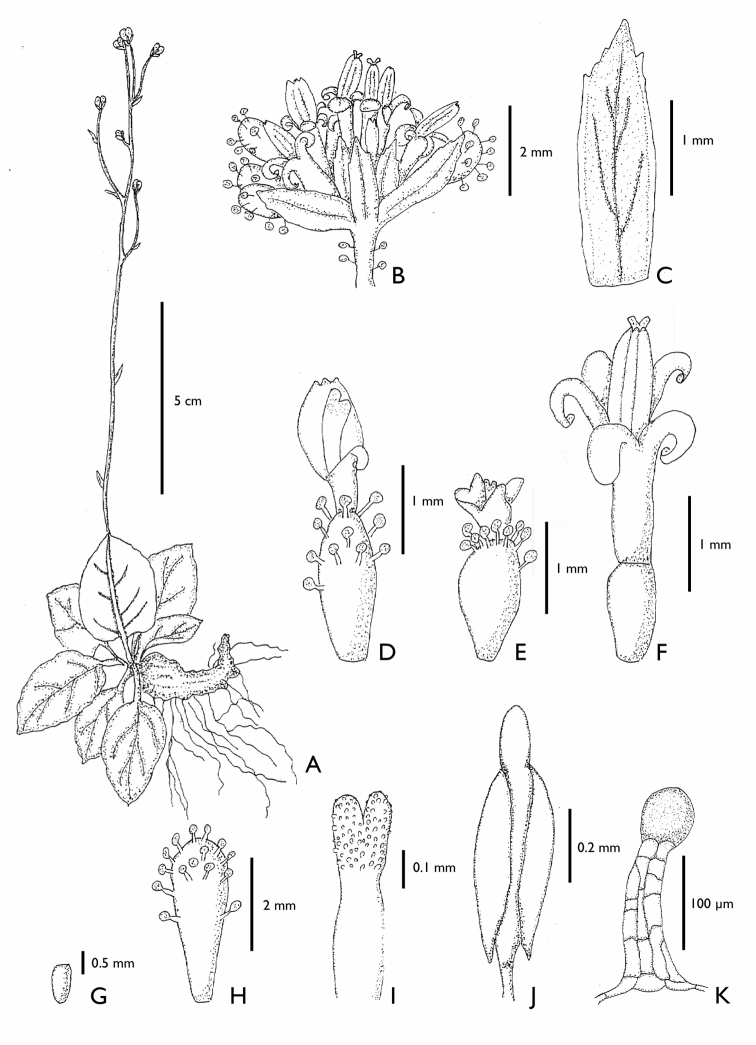
*Adenocaulon* (from Katinas 2000). **A** habit **B** head **C** involucral bract **D–E** marginal florets **F** central floret **G** central achene **H** marginal achene **I** upper part of style **J** stamen **K** achene hair multiseriate, capitate, glandular. (*Adenocaulon chilense*: **A** LP *s.n. ex* LPS *16554*; **B–D, F** *Cabrera* et al. *23066* LP; **G–H** *Ricardi* et al. *1983* LP. *Adenocaulon bicolor: E Hedgcock s.n.* LP, **I–K** *Morrison 121* LP)

*Eriachaenium* Sch. Bip. and *Adenocaulon* Hook. (Figs 1–5) are perennial herbs that were left unplaced in the most recent genus level classification of the Compositae family (Hind 2007). More recent phylogenies, based on molecular data, have divided the Compositae into 42–43 tribes; about half of them are small (Panero and Funk 2008; Funk et al. 2009). Within these family level phylogenies there are four main areas (as well as a number of very small subfamilies), beginning with the crown group: the highly nested subfamily Asteroideae (asters, ragworts, sunflowers: monophyletic) which contains about 65% of the species in the family; the subfamily Cichorioideae s.l. (gazanias, dandelions, ironweeds: monophyletic); the subfamily Carduoideae (thistles and African Mutisieae: monophyletic), and finally there is a paraphyletic basal area of the phylogeny that contains most of what used to be the tribe Mutisieae s.l. (Gerbera daisies) whose former members are now in 15 tribes (Funk et al. 2009). Throughout its history *Eriachaenium* has been placed in three tribes in the subfamily Asteroideae and one in the basal grade; *Adenocaulon* has previously resided in six tribes in the Asteroideae and two in the basal grade.

Unlike many taxa that are difficult to place, *Eriachaenium* is rarely discussed or debated. Perhaps it’s remote location (endemic to Patagonia; Fig. 3) or the fact that it is monospecific (*Eriachaenium magellanicum* Sch. Bip) and relatively rare, has fostered this lack of attention. This small herb has an unusual compressed underground stem and staminodes in the marginal florets (Figs 1, 2). The species was described by Schultz Bipontinus (1855) who placed it near *Osteospermum*, an African genus in an almost exclusively African tribe (Asteroideae: Calenduleae). Although today this placement is difficult to understand it was probably based on the long corolla lobes and short anther bases. Bentham (1873) and Hoffman (1894) did not disagree with this placement and there it remained until Cabrera (1961) in his key to the Argentine genera of Asteraceae moved it to the Inuleae (Asteroideae) without comment, but in the subtribe Adenocaulinae along with *Adenocaulon*. Gray described Adenocaulinae (1873) but included only the type. *Eriachaenium* was left in the Inuleae by Muñoz Pizarro (1966) as well as Moore (1983). Robinson and Brettell (1973) moved the genus to the Mutisieae s.l., based mainly on pollen characters and Bremer (1994) put the genus in the Mutisieae subtribe Nassauviinae (now tribe Nassauvieae). *Eriachaenium* was not included in recent molecular phylogenies such as Funk et al. (2005), Panero and Funk (2008), Funk et al. (2009, 2014).

*Adenocaulon* has five species that grow in temperate forests (Fig. 4A) in four widely disjunct areas (Fig. 3): Northwest USA and adjacent Canada (1 species), East Asia (2), Mesoamerica (1), and Patagonia (1) (Bittman 1990a, b). In contrast to *Eriachaenium*, *Adenocaulon* has received quite a bit of attention possibly because it is more widespread and most of its taxa are found in areas with numerous botanists. These various studies have moved the genus from tribe to tribe; in fact, over the years it has been placed in eight tribes. Along with a few other genera, *Adenocaulon* was unplaced to tribe by Bremer (1994). The various placements are as follows:

(1) Eupatorieae: Edgeworth (1851); (2) Heliantheae s.l. (in tribe Millerieae): Bentham and Hooker (1873), followed by Gardner (1977); (3) Inuleae: Gray (1873, as the separate subtribe Adenocaulinae), followed by Hoffmann (1894), Britton and Brown (1943), Cabrera (1961, 1971), Muñoz Pizarro (1966), Merxmüller (1977), and Moore (1983); (4) Adenocauleae: Rydberg (1917), monogeneric; (5) Senecioneae: Cronquist (1955), followed by Wagenitz (1964), and Morton (1978); (6) Anthemideae: Stix (1960), followed by Leins (1968), and Skvarla et al. (1977); (7) Mutisieae: Stebbins as quoted in Ornduff et al. (1967), followed by Nordenstam (1977), Bittman (1990a, b), Bremer (1994), Jansen and Kim (1996), Panero and Funk (2008); Katinas et al. (2008) and (8) Cardueae: Maximova (1999).

Cabrera (1961) and Katinas (2000) were the only ones to consider the two genera together. Cabrera (1961) published a key to the genera of Asteraceae of Argentina and, probably following the classification of Gray (1873) who placed *Adenocaulon* in the subtribe Adenocaulinae of the tribe Inuleae. Cabrera (1961) accepted that placement and also included *Eriachaenium* in the subtribe. Katinas (2000) conducted a cladistic analysis using 38 morphological characters and 52 genera from across the family to investigate whether or not *Adenocaulon* and *Eriachaenium* were closely related to one another and to determine a tribal assignment for the two genera. Her study showed that the two genera were closely related based on sharing four characters: 1) involucral bracts in 1–2 series, 2) length/width ratio of anthers was 2.5–4.5, i.e., very small; this ratio was only found in members of Anthemideae, 3) the lack of a pappus (characters that are found in other parts of the phylogeny), and 4) a re-occurrence of the plesiomorphic short bifid style. As far as placement in the family, Katinas results showed that ‘floret dimorphism’ and ‘pseudobilabiate florets shared with Anthemideae, *Adenocaulon* and *Eriachaenium* that are female or neuter and the tubular and/or pseudobilabiate florets’ place them above the Mutisieae and Cardueae. Also they share two synapomorphies, ‘floret dimorphism’ and ‘marginal florets female or neuter’, that group them with the Liabeae, Arctotideae, and Asteroideae. The placement of the two genera was determined to be (in todays classification) in the Cichorioideae (s.s.) above the Lactuceae/Vernonieae clade and the sister group of the Liabeae. This was yet another new position for both of these genera. *Adenocaulon* was included in the chloroplast DNA phylogenies of Panero and Funk (2008) and Funk et al. (2009) and placed in the Mutisieae s.s. but the generic representation from the Mutisieae s.s. was not extensive and *Eriachaenium* was not included.

In order to ascertain the best placement for *Eriachaenium* and *Adenocaulon* and to test the proposed sister group relationship between the two genera we used a molecular approach that included species of both taxa and a wide sampling of outgroups. In 2009, the members of a collecting expedition to Patagonia located populations of *Eriachaenium* and *Adenocaulon* (*Adenocaulon chilense* Less.) and this fresh material combined with two additional herbarium specimens (*Adenocaulon*: *Adenocaulon bicolor* Hook., and *Adenocaulon lyratum*) has allowed us to fully discuss these two genera and to provide an estimate on where they should be placed in the phylogeny of the family.

## Materials and methods

We sequenced the nuclear ITS and the plastid molecular markers *trnL-trnF* and *rpl32* of two different populations of *Eriachaenium magellanicum*, one each of *Adenocaulon chilense*, *Adenocaulon lyratum* and *Adenocaulon bicolor*, the sequences are deposited in GenBank and the numbers will be included in Pasini et al. (MS submitted). These data were shared with the authors of a separate study that encompassed a broad selection of taxa from the Mutisioideae and related tribes (Pasini et al. MS submitted) in order to determine the proper placement for these problematic genera. The molecular dataset contained species of the three tribes of the subfamily Mutisioideae (Mutisieae–8 genera, 21 species, Onoserideae–four genera, four species, and Nassauvieae–13 genera, 25 species) and four species of the subfamily Barnadesioideae. These data contain new sequences generated for the Pasini et al. (MS, submitted) as well as sequences from GenBank generated for several publications (Katinas et al. 2008; Simpson et al. 2009; Baird et al. 2010; Funk et al. 2014).

**Figure 6.**
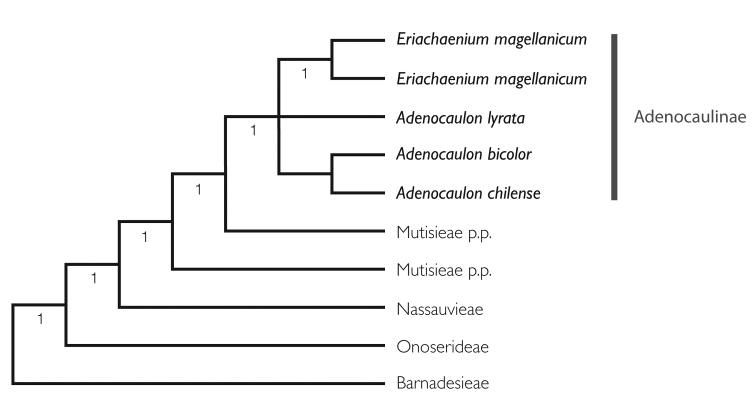
Simplified phylogeny showing placement of *Eriachaenium* and *Adenocaulon*.

Details of the sampling strategy, DNA extraction, amplification, and sequencing methods and data analysis discussion are included in the Pasini et al. paper (MS, submitted). Here we show a part of the final cladogram that highlights the placement of *Eriachaenium* and *Adenocaulon* (Fig. 6).

## Results and discussion

### Phylogeny

The results of all three datasets, the *matK*, the combined ITS-trnL-F, and the total combined show *Eriachaenium* and *Adenocaulon* forming a clade nested in the Mutisieae s.s. (Fig. 6). The support values of the clade formed by these two genera are high (Bayesian inference of 1) but the relationships among the three species of *Adenocaulon* sampled in the analysis and *Eriachaenium magellanicum* are not clear. Here we show a section of the phylogeny (Fig. 6) for the purpose of facilitating the discussion in this paper. For the complete phylogeny based on the combined ITS, *trnL-F*, *matK*, and *trnL-rpl32* markers see Pasini et al. (MS, submitted).

### Morphology

Most of the confusion in the placement of *Eriachaenium* and *Adenocaulon* is caused by a lack of understanding of character evolution within the family complicated by the fact that the characters that were most often used to define the Mutisieae s.l. are often missing or modified in both genera. Now that *Eriachaenium* and *Adenocaulon* form a clade nested in the tribe Mutisieae s.s. (Mutisioideae) we can re-examine the morphology of the two genera and how their characters fit with those of the Mutisieae s.s.

Prior to the advent of molecular data, the Mutisieae s.l. were considered to be highly derived because some were humming bird pollinated and many had some form of colorful and/or dimorphic corollas (especially bilabiate or pseudo-bilabiate), long tails on the anthers, short bifid styles often with a rounded apex, and psilate or microechinate pollen. Later characters such as anthemoid (ecaveate) pollen (Ornduff et al. 1967, Robinson and Brettell 1973), testa epidermis type (Grau 1980); chromosome number (n = 23; Ornduff et al. 1967), and “thickened apical appendage on anthers” and “obtuse-rounded style hairs” (Bremer 1994), were added to the list. These were thought to be derived characters because they were uncommon in the family and restricted, for the most part, to South American taxa, and because it was commonly believed by many taxonomists that studied the Compositae that the Heliantheae s.l. represented the ancestral morphology. Since the groundbreaking work of Jansen and his co-authors (Jansen et al. 1987, 1990, 1991, 1996) and subsequent contributions by Panero and Funk (2008) and Funk et al. (2009) we now know that the Mutisieae s.l. are actually a number of independent lineages strung out along the basal area of the phylogeny; some are even part of the thistle subfamily (Carduoideae) or independent lineages (Pertyeae; Pertyoideae).

Within the latest classification the tribe Mutisieae s.s. falls into the subfamily Mutisioideae with two additional tribes: Onoserideae, Nassauvieae. This more restricted version of the tribe is defined by the presence of many of the same characters mentioned above because many of the taxa that lack those characters are now placed elsewhere. However, as Katinas et al. (2008) correctly pointed out, some of these characters are found in other tribes. *Eriachaenium* and *Adenocaulon* have some of these characters (dimorphic corollas; short bifid styles with a rounded apex; microechinate, ecaveate pollen) but lack others (colorful corollas, long tails on anthers). While *Adenocaulon* has bilabiate corollas, *Eriachaenium* does not, however it does have a variable number of corolla lobes (4 or 5) so technically they both have dimorphic corollas.

### Pollen and chromosome numbers

The pollen grains of *Eriachaenium* and *Adenocaulon* (Fig. 6) are very similar to one another differing only in the size of the grain and the thickness of the exine and both genera have some grains with a compact aspect and Anthemoid pattern (see also Skvarla et al. 1977; Bittmann 1990a). A study by Zhao et al. (2006) has the only SEM images that show the exine structure that we know of for both genera and they appear to be of the standard pollen type for the Mutisioideae. *Adenocaulon* and *Eriachaenium* have pollen of medium size (P × E = 26–32 × 26–30 µm in *Adenocaulon*, and 30–36 × 24–30 µm in *Eriachaenium*), are tricolporate, with the colpi long with thin margins and a microgranulate membrane and a diffuse mesoaperture (Katinas et al. 2008). Both genera have a *Mutisia* type exine (Tellería et al. 2003). Overall the pollen grains of both genera are similar to that of *Artemisia verlotiorum* (Anthemideae) a condition that exemplifies one of the major problems with trying to identify unique morphological characters to define groups within the family (Hansen 1991). Ornduff et al. have a very interesting quote in their 1967 paper (page 212):

“We are not convinced that *Adenocaulon* belongs in Senecioneae where it has been placed by various workers (Ornduff et al. 1963), but the count of n = 23 for the very local and distinctive Central American *Adenocaulon lyratum* is a report for the fourth … member of the genus to be examined. Each species has consistently had n = 23.… Stebbins (personal communication) has suggested that *Adenocaulon* shows affinities with Mutisieae on the basis of a common possession of distinctive pollen characters. The bilabiate tendencies of marginal corollas, the shape and pubescence of leaves, and the chromosome number of *Adenocaulon* further suggest relationships to Mutisieae….”

### Taxonomy

#### Subtribe Adenocaulinae

Taxon classificationPlantaeAsteralesAsteraceae

Adenocaulinae A. Gray, Syn. Fl. N. Amer. 1(2): 59. 1884 (as “Adenocauleae”). TYPE: Adenocaulon Hook.

##### Description.

*Herbs* perennial, dwarf or scapiform with cylindrical or planate rhizomes, stems simple, erect or prostrate to ascending, glabrous or with stipitate-glandular hairs. *Leaves* glabrous to subglabrous above, tomentose beneath; basal leaves alternate or rosulate to sub-rosulate; sessile or petiolate to pseudopetiolate; blades oblanceolate, elliptic, ovate, obovate, to deltoid, margin entire to lyrate, pinnately or palmately veined, glabrous to subglabrous above, tomentose beneath. *Inflorescences* terminal or axillar, monocephalous or laxly racemose to corymbose, pedunculate; heads heterogamous, disciform; receptacle epaleate; involucre uniseriate. *Florets* dimorphic; marginal florets female, with or without staminodes, corolla sub-bilabiate (3+1 corolla lips), tubular-funnelform, shortly to deeply 4- to 5-lobed, rarely bilabiate; central florets bisexual or male with a rudimentary ovary, corolla tubular-funnelform, deeply 5-lobed; anther apical appendages rounded to acute at the apex, basally constricted and demarcated from the thecae, basally auriculate with tails very short, smooth to slightly papillose, filament with anther collar; style shortly bifid, branches dorsally papillose. *Achenes* truncate at the apex, densely pubescent, shaggy (long, filiform, uniseriate hairs) or glandulose (glandular multiseriate capitate hairs), dimorphic, marginal cypselae conspicuously bigger than the central ones; pappus absent. Pollen spheroidal to prolate, tricolporate, exine *Mutisia* type, microechinate.

The subtribe Adenocaulinae was described by Gray (1873), on the basis of the genus *Adenocaulon*, for the tribe Inuleae. Rydberg (1917) raised the subtribe to the independent tribe Adenocauleae, also with *Adenocaulon* as its only genus. Further, Cabrera (1961) returned to Gray’s concept and re-described the subtribe Adenocaulinae for the tribe Inuleae, but this time the subtribe included the genera *Adenocaulon* and *Eriachaenium*. Despite the addition of *Eriachaenium*, no emendation of Gray’s subtribe concept is needed because the short and general description of Gray includes the features common to both genera.

### Key to genera

**Table:** 

1	Herbs prostrate to ascending with leaves alternate; blades oblanceolate. Heads solitary. Achenes shaggy, covered by long, filiform hairs	***Eriachaenium***
-	Herbs scapiform with leaves rosulate; blades elliptic to deltoid. Heads laxly racemose or corymbose. Achenes covered by glandular hairs	***Adenocaulon***

#### Eriachaenium

Taxon classificationPlantaeAsteralesAsteraceae

Sch. Bip.

Figures 1 , 2

Eriachaenium Sch. Bip. Flora 38: 120. 1855. TYPE: *Eriachaenium magellanicum* Sch. Bip.

##### Etymology.

From the Greek *erion*, wool, and the Latin *achaenium*, a type of fruit, describing the villose fruits.

##### Description.

*Herbs* perennial, dwarf, with stout, oblique to vertical rhizomes that are compressed laterally, stems prostrate to ascending. *Leaves* alternate; sessile, clasping; blades oblanceolate, pinnately veined, margin entire to undulate-dentate, glabrous to subglabrous above, tomentose beneath. *Inflorescence* monocephalous, axillar; heads pedunculate, heterogamous, disciform; receptacle epaleate; involucre uniseriate. *Florets* dimorphic; marginal florets female with staminodes, corolla tubular-funnelform, deeply 4-lobed; central florets bisexual or male with a rudimentary ovary, corolla tubular-funnelform, deeply 5-lobed; anther apical appendages rounded to acute, basally constricted and demarcated from the thecae, anthers dark, basally auriculate with tails very short, smooth to slightly papillose; style bilobed, dorsally papillose. *Achenes* truncate at the apex, densely pubescent, dimorphic, the marginal achenes conspicuously bigger than the central ones; pappus absent. [modified from Katinas et al. 2008]

Pollen spheroidal to prolate, spheroidal or elliptic in equatorial view, circular in polar view, medium size, P × E = (30–36 × 24–30) µm. Tricolporate, colpi long with thin margin and microgranulate membrane, mesoaperture diffuse. Exine *Mutisia* type, microechinate, 2–6 µm thick, slightly slender at the poles. Ratio ectosexine/endosexine: 1:1.5; 1:2. Nexine 1.5 µm thick. SEM: tectum punctate.

Habitat and distribution. Genus with only one species, *Eriachaenium magellanicum* Sch. Bip., endemic to Patagonia in Argentina and Chile (Fig. 3). It grows in mud, sand, and pebbles either along the margins of inland somewhat saline lakes or near the coast in estuaries (Morore 1983 and field observations).

##### Species list.

*Eriachaenium magellanicum* Sch. Bip., Flora 38: 121. 1855.

#### Adenocaulon

Taxon classificationPlantaeAsteralesAsteraceae

Hook., 1829

Figures 4 , 5

Adenocaulon Hook., Bot. Misc. 1: 19. 1829. TYPE: *Adenocaulon bicolor* Hook.

##### Etymology.

From the Greek *aden*, gland, and *kaulos*, stalk, stem, describing the stalked glandular hairs.

##### Description.

*Herbs* perennial, scapiform with stout rhizomes, stems simple, erect, with stipitate-glandular hairs. *Leaves* glabrous to subglabrous above, tomentose beneath; basal leaves rosulate to sub-rosulate; petiolate to pseudopetiolate; blades elliptic, ovate, obovate, to deltoid, margin entire to lyrate, pinnately to palmately veined; upper leaves similar to the basal ones but few and reduced. *Inflorescence* terminal, laxly racemose to corymbose, on long peduncles; heads pedunculate, heterogamous, disciform; receptacle epaleate; involucre uniseriate. *Florets* dimorphic; marginal florets female, without staminodes, corolla sub-bilabiate (3+1 corolla lips), tubular-funnelform 4- to 5-lobed, rarely bilabiate; central florets male with a rudimentary ovary, corolla tubular-funnelform, deeply 5-lobed; anther apical appendages rounded to acute at the apex, basally constricted and demarcated from the thecae, anthers light colored and basally auriculate with tails very short, smooth; style bilobed, branches dorsally papillose. *Achene* truncate at the apex, pubescent (glandular multiseriate capitate hairs), dimorphic, marginal achenes conspicuously bigger than the central ones; pappus absent. [modified from Katinas et al. 2008]

Pollen spheroidal, circular in polar view, medium size, P × E = (26–32 × 26–30) µm. Tricolporate, colpi long with thin margin and microgranulate membrane, mesoaperture diffuse. Exine *Mutisia* type, microechinate, 4–5 µm thick, slightly slender at the poles. Ratio ectosexine/endosexine: 1:1.5; 1:2. Nexine 1.5 µm thick. SEM: tectum punctate. Note: pollen of *Adenocaulon bicolor* was found to be identical to that of *Adenocaulon chilense* Less.

Habitat and distribution: Genus of five species with a disjunct distribution in Patagonia, Mesoamerica, northern United States and southern Canada, and temperate southeastern Asia (Fig. 3). Inhabits moist forests in the shade of *Pinus* spp., *Quercus* spp. and *Nothofagus* spp. (Bittmann 1990a, b, and field observations). Details of the flower morphology, including the differences between the male and female flowers, can be found in Ayers (1900).

##### Species list:

five species falling into three morphological groups that are biogeographically distinct (according to Blake 1934):

**Group A: North America and East Asia**

1. *Adenocaulon bicolor* Hook., Bot. Misc. 1: 19. 1830. (British Colombia to south central California, eastward to Montana and sparingly to Michigan)

*Adenocaulon integrifolium* Nutt.

2. *Adenocaulon himalaicum* Edgew., Trans. Linn. Soc. London 20: 64. 1851. Himalayan region and Japan

*Adenocaulon adhaerescens* Maxim. (described from Japan)

3 *Adenocaulon nepalense* M. Bittmann, Candollea 45: 403. 1990. Nepal

**Group B: Chiapas, Mexico & Guatemala**

4. *Adenocaulon lyratum* S. F. Blake, J. Wash. Acad. Sci. 24: 435 1934.

**Group C: South America**

5. *Adenocaulon chilense* Less., Linnaea 6: 107. 1831. (Southern Chile and the Magellan region)

*Adenocaulon lechleri* Sch. Bip.

## Conclusion

Perhaps the best conclusion is to review synapomorphies for the *Eriachaenium + Adenocaulon* clade. With the phylogeny available we can examine the characters that group the two genera. That does not mean that none of the other species in the family or even in the Mutisioideae have these characters, it means that, when examined in the light of the phylogeny they are deemed synapomorphies for the *Eriachaenium + Adenocaulon* clade.

1. Within the Mutisioideae s.s. the tails are short only in *Adenocaulon* and *Eriachaenium* (Figs 2J, 5J).

2. Within the Mutisieae s.s. the anther collar is only found in *Adenocaulon* and *Eriachaenium*.

3 & 4. Two synapomorphies that are most likely linked are the dimorphic florets and achenes: the marginal, functionally female florets (Figs 2D, E, 5D, E) have larger achenes (Figs 2D, G, 5D, G) and the central, functionally male florets (Figs 2F, 5F) have smaller achenes (Figs 2H, 5G).

5. It is interesting to note that in these two genera the florets’ dimorphism is not conspicuous, while in all the other genera of the tribe it is. In fact, within the Mutisieae s.s. there is an impressive variety of colors of the marginal florets which easily distinguishes them from the central florets. Therefore the character of “inconspicuously dimorphic florets” found in the *Eriachaenium + Adenocaulon* clade and not found in the Mutisieae s.s., can also be considered as a synapomorphy.

6. The presence of tubulose 4-lobed corollas in the marginal florets in both genera indicates a strong affinity between the two genera because while tetramerous central florets are common in Compositae, such corollas rarely occur as marginal ones.

7. Even though *Eriachaenium* and *Adenocaulon* have a *Mutisia* exine type of pollen, the grains are small and spheroid with a thin exine, whereas those of Mutisioideae (excluding Nassauvieae) are usually large and elliptic with a thick exine. This type of pollen grain is unique in the Mutisieae and it approaches the *Artemisia* exine type (Anthemideae).

8. Both genera lack a pappus (Figs 2D–H, 5D–H). The absence of a pappus is widespread in other tribes of Compositae (e.g., Heliantheae s.s.) but it is very rare in Mutisioideae (only found in Adenocaulinae and *Cephalopappus* and *Panphalea* of the tribe Nassauvieae).

9. Both genera have their achenes covered with unusual pubescence: *Eriachaenium* has multicellular, flagellate, filiform hairs that are confined to the achene (Fig. 2D, F–H, K) and *Adenocaulon* has multiseriate, capitate, glandular pubescence (the glands are dark purple) and this pubescence is found on other parts of the inflorescence (Figs 4D, E, 5B, D, E, H, K).

10. *Eriachaenium* and *Adenocaulon* both grow in habitats that are unusual for the family: *Eriachaenium* practically buries itself in the sandy mud (Fig. 1B, E) and when we found it, it was a few feet above the water line of some, but certainly not all, lakes/ponds in the mountains of Patagonia (Fig. 1A); *Adenocaulon* inhabits the floor of relatively moist forests (Fig. 4A–C). Both of these are rather extreme limits of the habitat for the family. Perhaps this movement into these habitats has triggered their unusual morphology.

Another potential synapomorphy is the bullate leaves found in both genera. But, more data need to be gathered to be sure of its distribution in the Mutisioideae.

We can also list some characters that we now think are plesiomorphic for the *Eriachaenium* + *Adenocaulon* clade in that they are shared with other parts of the basal grade: central corollas deeply lobed; style shortly bifid with an apex that is rounded or slightly acute; style apex shortly papillose; pollen with exine psilate or microechinate and pollen of the Anthemoid pattern. The pollen grains in *Eriachaenium* and *Adenocaulon* share features with many taxa in the Mutisioideae and with Anthemideae. However, at this point, the occurrence of the “anthemoid” pollen in the Anthemideae is considered to be independent of its occurrence in the Mutisioideae.

In future studies we hope to expand these lists as well as determine the point on the cladogram where the plesiomorphic characters are actually apomorphic.

## Supplementary Material

XML Treatment for Adenocaulinae

XML Treatment for Eriachaenium

XML Treatment for Adenocaulon
